# Lights and shadows of artificial intelligence in laboratory medicine

**DOI:** 10.1515/almed-2025-0024

**Published:** 2025-02-24

**Authors:** Giuseppe Lippi, Mario Plebani

**Affiliations:** Section of Clinical Biochemistry and School of Medicine, University of Verona, Verona, Italy; Department of Laboratory Medicine, University of Padova, Padova, Italy

**Keywords:** artificial intelligence, laboratory medicine, deep learning, large language models

Although a universally accepted definition does not exist, artificial intelligence (AI) is commonly described as the use of computational systems to simulate human intelligence, with the purpose of performing a vast array of tasks that require reasoning, learning and decision-making [1]. The term “artificial intelligence” was originally coined by John McCarthy and colleagues in the mid-1950s, when it was defined as “the science and engineering of making intelligent machines that exhibit critical thinking comparable to humans” [2]. Since then, remarkable technological advancements have enhanced the power and use of AI tools, making them an integral part of both personal and professional domains. Key components of AI include machine learning (ML), which encompasses algorithms designed to improve autonomously through experience, and deep learning (DL), a subset of ML that uses neural networks with multiple layers to analyze complex data. Neural networks themselves are computational models that replicate the structure of the human brain, enabling capabilities such as pattern recognition and predictive modeling [3]. Generative AI, a specific subset of AI, uses DL models, and is hence primarily focused on generating novel content, including text (e.g., large language models, LLMs), images, music and so forth [3].

In healthcare, AI systems have been developed to improve efficiency and accuracy, particularly in tasks such as diagnosing diseases from imaging data and processing large amount of clinical and diagnostic information [4]. Due to the extensive use of technology and the potential to generate an immense amount of clinical information (commonly referred to as “big data”) in the form of laboratory test results, AI has gained significant momentum in the field of laboratory medicine [5]. A recent survey conducted by the European Federation of Clinical Chemistry and Laboratory Medicine (EFLM) revealed considerable interest in AI training among laboratory professionals, even if only a minority (approximately 25 %) of surveyed laboratories reported having active AI projects in place [6].

Regardless of the varying perceptions among laboratory professionals, it is indisputable that AI will continue to play an increasingly prominent role in organization and operations of medical laboratories. Several AI tools are already widely deployed in clinical laboratories, such as ML models for optimizing sample processing and quality control management [7], automated verification and validation modules integrated with the laboratory information system (LIS) [8], digital morphology techniques in laboratory hematology [9] and for assisting diagnostic reasoning across various medical fields [10], as well as tools for supporting creation and editing of documents. Additionally, DL models are playing an essential role in managing and interpreting the massive datasets generated through genomics and proteomics research.

A paradigmatic example of how AI can support clinical decision-making has been provided by a recent single-blind randomized clinical trial, in which physicians specializing in family, internal or emergency medicine were randomized to either use conventional resources alone or to have access to the same resources supplemented with LLM [10]. Although the use of LLM did not significantly improve clinical reasoning compared to the use of conventional resources alone (median diagnostic reasoning score per case: 76 vs. 74 %; p=0.60), the median diagnostic reasoning score for LLM alone was 92 %, thus offsetting the scores of both physician cohorts by over 16 %.

Although it is increasingly clear that AI will become an integral part of organization and activities of medical laboratory services worldwide, significant challenges remain (Table 1). The primary issue is the limited flexibility of AI in interpreting laboratory data. In contrast to human cognition, which can use intuitive judgment and gestalt reasoning [11], AI systems currently lack the adaptive capacity to interpret data with equivalent contextual understanding. The same principle applies to image recognition. While DL models can be trained to identify digital images of prototype cell types or other components in body fluids, the inherent heterogeneity in the presentation of these elements – especially the variability of abnormalities across different physiological or pathological states – limits the accuracy of classification. This challenge persists whether using specialized digital cell imaging systems or more general AI tools, like ChatGPT [12]. In all these cases the current generation of AI tools would still need interactive learning frameworks and human oversight for reviewing the initial classification. Analogously, the interpretation of human symptoms and individual laboratory data by AI often relies on predefined algorithms that are designed to identify patterns based on existing data and conditions. Nevertheless, diseases can present in many diverse ways, and the range of possible laboratory abnormalities associated with different conditions can vary widely, making it challenging to always provide accurate or comprehensive interpretations [5], 13]. Additional limitations include the potential for overreliance on AI systems, which could completely replace human reasoning; limited flexibility and adaptability, leading to errors in interpreting complex or rare laboratory abnormalities, as previously exemplified; the lack of standardization, as different AI tools may yield inconsistent answers to the same clinical question [14]; the regulatory challenges that must drive integration into routine laboratory practice; the inherent cost of acquiring and regularly updating the software; the complexity between human and AI interaction; the lack of transparency and explainability due to the often unidentified nature of the algorithms; accountability concerns when errors occur; ethical and legal issues related to privacy, data security and potential bias in decision-making [15]. Finally, the use of an external validation set is always needed to ensure reproducibility and generalizability of any AI tool or algorithm.

**Table 1:** The lights and shadows of artificial intelligence (AI) in laboratory medicine.

Advantages
– Possibility to streamline process and activities by automating routine tasks – Autoverification and autovalidation of laboratory data, enhancing efficiency and accuracy – Assistance in digital image recognition for improved diagnostic accuracy – Support in interpretation of laboratory data, aiding clinical decision-making – Facilitated creation and editing of documents – Management and analysis of big data

**Limitations**

– Limited flexibility and adaptability in handling unexpected or complex data – Potential errors in interpreting complex or rare laboratory abnormalities – Overreliance on AI, potentially diminishing human clinical judgment – Lack of standardization, leading to inconsistent results between different AI tools – Regulatory requirements, which may slow down AI integration – High costs related to setup, maintenance and training for AI systems – Complexity in human–AI interaction – Lack of transparency and exploitability in AI decision-making processes – Poor accountability for errors made in data interpretation – Ethical and legal concerns, including data privacy issues and algorithmic bias – Need for external validation to ensure reproducibility and generalizability

In conclusion, while AI holds promise to revolutionize laboratory medicine by enhancing efficiency, diagnostic accuracy and data management, it also carries significant challenges. The integration of AI into routine laboratory practice must be approached with caution, addressing key unresolved issues such as risk of overreliance, limited flexibility and potential for bias and errors. Regulatory, ethical and legal concerns must also be addressed to ensure that AI is deployed transparently and accountably. As AI continues to evolve, its role in laboratory medicine services will expand, but its successful implementation will require active collaboration among laboratory professionals, technology developers, policymakers and healthcare managers.
